# Simultaneous Schlieren-Shadowgraph Visualization and Temperature Measurement Fields of Fluid Flow Using One Color CCD Camera

**DOI:** 10.3390/s22239529

**Published:** 2022-12-06

**Authors:** Adrián Martínez-González, David Moreno-Hernández, Miguel León-Rodríguez, César Carrillo-Delgado, Enrique Cossío-Vargas

**Affiliations:** 1 Departamento de Ingeniería Robótica, Universidad Politécnica del Bicentenario, Carr. Silao—Romita Km 2, San Juan de los Duran, Silao 36283, Guanajuato, Mexico; 2 Centro de Investigaciones en Óptica A.C., Loma del Bosque 115, Lomas del Campestre, León 37150, Guanajuato, Mexico; 3 Departamento de Ingeniería Robótica, Universidad Politécnica de Guanajuato, Av. Universidad sur 1001, Cortázar 38496, Guanajuato, Mexico; 4 Cuerpo Académico de Ingeniería en Agrotecnología, Universidad Politécnica del Bicentenario, Carr. Silao—Romita Km 2, San Juan de los Duran, Silao 36283, Guanajuato, Mexico

**Keywords:** Schlieren techniques, shadowgraph, measure temperature, crosstalk

## Abstract

Visualizing two variables in the fluid flow requires a complex optical system to fulfill such a purpose. Furthermore, in these applications, measuring the temperature fields of the fluid is sometimes necessary. In this work, we use a color digital camera to record in one snapshot shadowgraph and two direction sensitivity Schlieren images. Furthermore, the Schlieren images obtained are used to measure temperature fields. The optical system, as main components, employs an *rgb* Light Emitting Diode (LED), two knife edges represented by shortpass and longpass ultra-thin filters, and a color digital camera. The results of this study show that shadowgraph images are contaminated with the crosstalk effect, and correction against it was applied. On the other hand, the reached resolution of the optical system to measure temperature values is approximately one degree.

## 1. Introduction

Detailed visualization of the fluid flow behavior is of great importance in various branches of science [1,2,3,4,5]. On the other hand, temperature measurement fields in fluid flow are essential in several applications [6,7,8,9,10]. In addition, in some studies, it is necessary to detect two physical variables due to the phenomenon’s physical nature. Therefore, the data obtained in these optical systems are relevant since the phenomenon under study is described with greater certainty [11].

For those reasons, several techniques have been developed to fulfill such a purpose, mainly optical techniques. The main advantages of the optical methods are that they are full-field and non-intrusive [12]. Among the most important visualization techniques are the shadowgraph and Schlieren methods. These methods are easy to implement and use standard optical components. Moreover, they have high and variable sensitivity [13,14]. For example, the shadowgraph and the Schlieren methods detect displacement and deflection of the light rays due to changes in the refractive index. In a shadowgraph image, these displacements are represented as shades on the observation plane, and the technique’s sensitivity is in the horizontal and vertical directions. However, in a Schlieren method, its sensitivity is only in one direction; it depends on the knife edge’s orientation in the optical system. Therefore, a Schlieren system does not provide complete information on the fluid flow under analysis. As a result, several works have been proposed to solve this drawback using different optical setups [15,16,17,18,19,20,21,22].

Nevertheless, some of the optical systems developed are complex since they require complicated alignments due to the number of components and may be costly [15,16,17,18,19]. In other approaches where the optical arrangement is simple, image resolution may be compromised due to diffraction effects because of the size of the source filter and aperture [20,21]. Moreover, the optical system uses a halogen lamp in which the sensor receives non-filtered spectrum wavelength components causing a low image quality [21]. In other studies, the refractive index gradients are determined by relating ray deflection to color attribute variations (hue intensities) of the Schlieren images [22]. Of these reported works, some are used only for visualization, and others to determine temperature fields. However, none of these studies reported the determination of two variables (displacement and deflection of light rays). In [11], the Schlieren and shadowgraph techniques were used to detect the displacement and deflection of light rays. However, the used Schlieren optical system was sensitive only to one direction, and the complete optical system is complex to implement.

Recently, another simple alternative was proposed [23]. In [23], the optical system allows for simultaneous displacement and deflection detection of the light ray deviations due to changes in the refractive index. Moreover, the ray deflection is obtained in horizontal and vertical directions. In addition, the fluid flow temperature field is calculated using the Schlieren images. Consequently, this approach provides a complete visualization and measurements of temperature fields of fluid flow. However, the obtained shadowgraph images are contaminated with the crosstalk effect, and the temperature resolution value reported is limited for some applications. The crosstalk effect occurs when sensibilities overlap on the sensor and distort the recorded data, forcing compensation algorithms to use [24]. In addition, an increase in the Schlieren sensitivity system is necessary to have a reasonable temperature resolution value.

In this way, the main objective of this work is to correct the shadowgraph images against crosstalk by implementing algorithms for this purpose. In addition, increase the sensitivity of the Schlieren system to improve the temperature resolution value. The temperature measurement test will be achieved by conducting controlled experiments measuring temperature profiles of the convective fluid flow generated by a heated rectangular metal plate. These improvements in the results allow the analysis of fluid flow phenomena with high reliability. Therefore, the optical setup implemented is a z-Schlieren arrangement with two independent knife edges in the same beam path. The knife edges correspond to shortpass and longpass ultra-thin filters [25]. The filters are used to enhance vertical and horizontal refractive index variations in Schlieren images. The Schlieren and shadowgraph images are recorded simultaneously in an *RGB* digital camera.

## 2. Schlieren and Shadowgraph Techniques

The optical methods sensitive to fluid flow density variations studied in this work are the Schlieren and shadowgraph. This study uses these two techniques to visualize and measure temperature fields. The Schlieren technique requires a calibration procedure to measure temperature fields [8,9]. In these optical techniques, we assume that the density and the refractive index of the fluid are linearly related through the Gladstone–Dale equation [12]:

(1) (n−1) = Kρ

where *ρ* and *n* are the density and the refractive index of the gas, and *K* is the Gladstone–Dale’s constant. The Gladstone–Dale’s constant is a function of both the light source’s wavelength and the gas’s physical properties [12]. Each technique registers only one variable in the observation plane, i.e., ray displacement and deflection for shadowgraph and Schlieren, respectively. Naturally, every optical system has specific characteristics to fulfill such a purpose.

*Shadowgraph method.* Fluid flows with varying density changes when light rays pass through it, causing an intensity change in the observation plane that manifests as shadows. These intensity variations are proportional to the second derivative of the density [12,13]:

(2) $$\frac{\Delta I}{I}\approx lK\int_{0}^{h}\left(\frac{\partial^{2}\rho }{\partial^{2}x}+\frac{\partial^{2}\rho }{\partial^{2}y}\right)$$

where *I* is light intensity, *x* and *y* are cartesian coordinates perpendicular to ray propagation, and *h* and *l* represent the width and position of the object under test. The system’s sensitivity can be manipulated by changing the value of the object’s position under analysis.

*Schlieren method.* In Schlieren visualization, the integral flow properties along a given ray’s optical path contribute to the light intensity at the observation plane. In a classical Schlieren system, a primary lens is used to collimate a light source; the collimated light passes through the working section and is later refocused by a second lens. In the focal point of the second lens, a knife edge is placed to block part of the incident light. The knife edge blocks light rays from negative density gradients and allows the passing of the light rays from positive density gradients. The positive and negative density gradients in the observation plane are visualized as light and dark, respectively. In addition, the detection of the density gradient’s orientation is determined by the direction of the knife edge position (horizontal or vertical). In addition, the sensitivity of a Schlieren optical setup is determined by the focal distance of the second lens and the location of the knife edge device with respect to the image of the light source. A profound explanation of the Schlieren technique is found in [12,13].

The density gradient in a typical Schlieren method is obtained in only one direction; it depends on the orientation of the knife edge at the focal point of the second lens. Some modifications in the optical system are necessary to determine density gradients in both directions simultaneously. That is, its magnitude is determined in a predetermined direction, such as:

(3) $$\rho_{x}\;=\;\frac{\partial \rho }{\partial x}\;=\;\frac{\delta x}{f_{2}hK},\rho_{y}\;=\;\frac{\partial \rho }{\partial y}\;=\;\frac{\delta y}{f_{2}hK}$$

where *h* is the width of the convective fluid flow under test, and *f*_2_ is the focal distance of the second mirror. Therefore, calculating the density gradients requires knowing the value of *δx* and *δy*, which in this work can be obtained using calibration curves.

## 3. Cross Talk Correction

A digital color camera records *RGB* images that can be separated into their respective color channel. Each channel corresponds to the red (*R*), green (*G*), and blue (*B*) colors and provides particular information about the object under study. In these cameras, the green color channel is more sensitive than the other two color channels because it is configured as a Bayer filter mosaic. On the other hand, the incident light on pixels of each color channel is expressed with the following equation [25]:

(4) $${\hat{I}}_{i}\;=\;\sum_{j=0}^{2}A_{ij}I_{j}$$

where $A_{ij}$ is the crosstalk coefficient that relates the color spectral response of the camera pixels and incident illumination. The *i* and *j* subscripts denote the color channel and incident illumination, respectively. In addition, ${\hat{I}}_{i}$ represents the intensity projected in each color channel. The light intensity variable $I_{j}$ is not known and is needed to calculate solving Equation (4). Hence, the formula (4) can be denoted in the following way:

(5) $${\hat{I}}_{R}\;=\;A_{Rr}I_{r}\;+\;A_{Rg}I_{g}\;+\;A_{Rb}I_{b}$$

(6) $${\hat{I}}_{G}\;=\;A_{Gr}I_{r}\;+\;A_{Gg}I_{g}\;+\;A_{Gb}I_{b}$$

(7) $${\hat{I}}_{B}\;=\;A_{Br}I_{r}\;+\;A_{Bg}I_{g}\;+\;A_{Bb}I_{b}$$

The crosstalk coefficients represented in Equations (5)–(7) are calculated using the spectral response of color channel pixels and incident illumination. The matrix form of Equations (5)–(7) is expressed as follows:

(8) $$\left[\begin{array}{c} {\hat{I}}_{R}\\ {\hat{I}}_{G}\\ {\hat{I}}_{B} \end{array}\right]\;=\;\left[\begin{array}{ccc} A_{Rr} & A_{Rg} & A_{Rb}\\ A_{Gr} & A_{Gg} & A_{Gb}\\ A_{Br} & A_{Bg} & A_{Bb} \end{array}\right]\left[\begin{array}{c} I_{r}\\ I_{g}\\ I_{b} \end{array}\right]$$

Finally, determining the incident light in each color channel free of the crosstalk effect requires the solution of the previous equation.

## 4. Experimental Setup

**Figure 1:**
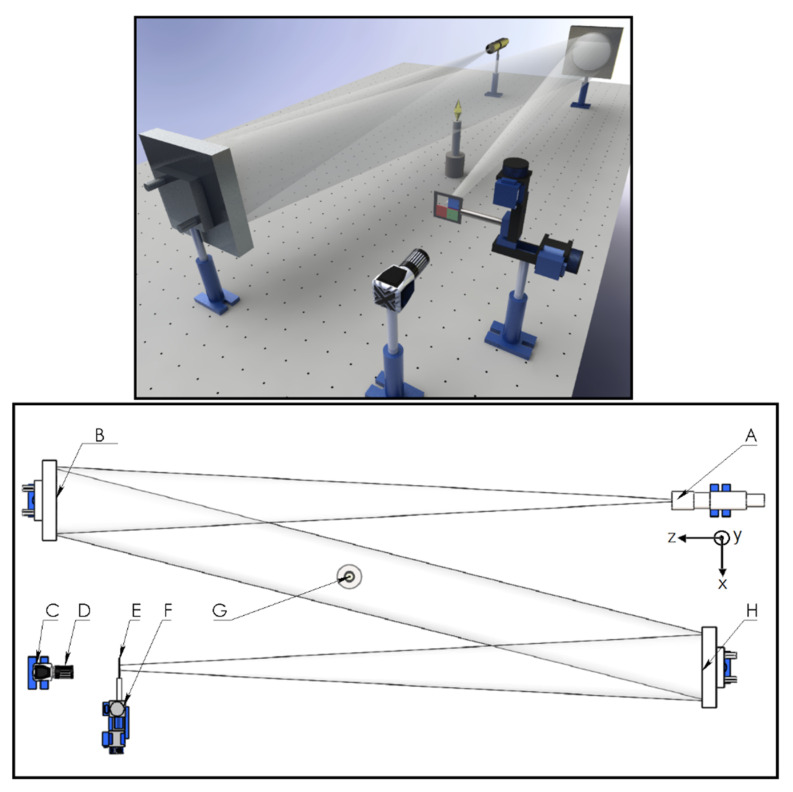
Scheme of horizontal-vertical sensitivity Schlieren system and shadowgraph. A—*rgb* LED, B,H—Mirrors, C—Camera, D—Lens 50mm, E—Shortpass and longpass filters, F—Motorized translation stage, G—Sample test.

The optical setup consists of two spherical mirrors of focal distance *f* = 1.54 m, an illumination *rgb* Light Emitting Diode (LED), an ultra-thin shortpass filter, an ultra-thin longpass filter, and an *RGB* color digital camera (see Figure 1). The *rgb* LED model WS2812B is manufactured by Worldsemi and includes a display unit that allows us to control it. In addition, the camera used is a Lumenera Lt225c model (2.2 Megapixel CMOS sensor/5.5 µm pixel size).

The *rgb* intensity light collimated by a first mirror is incident on a second mirror, and the light is reflected in its focus. The two ultra-thin filters are located at the focal point. Each filter allows approximately 40% of the light on it to pass through. The shortpass and longpass filters are positioned at the focal point horizontally and vertically, respectively. Finally, the object under analysis is imaged on a color digital camera using a lens with a focal distance of 50 mm.

**Figure 2:**
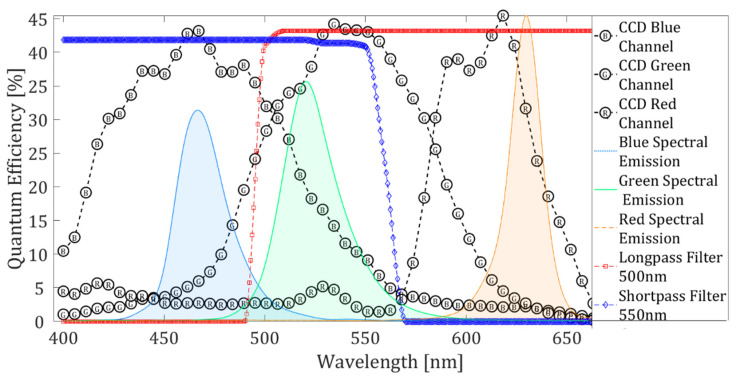
Relation between the spectral responses of the color digital camera, *rgb* LED, and shortpass and longpass filters.

The spectral responses of the shortpass and longpass filters, pixels of the digital camera, and the *rgb* illumination are shown in Figure 2 [24,26]. The *rgb* illumination spectral response is obtained using an Ocean Optics mini-spectrometer model HR4000. Figure 2 shows that the *rgb* spectrum is formed of three peaks corresponding to red, green, and blue colors. Unfortunately, the green peak overlaps with greater intensity in the blue and green channels of the digital camera, and correction against this effect is required (see Section 3). On the other hand, we can notice the impact of these three lighting peaks in the presence of the filters. Note that the filters do not block only green light. However, blue and red lights are blocked by the longpass and shortpass filters, respectively.

The tests are achieved by visualizing the convective fluid flow generated by a pencil soldering iron flame. In addition, the temperature fields are calculated from the convective fluid flow generated on a heated rectangular metal plate. The plate (7.3 cm × 11 cm) used in this work is a controlled chilling/heating plate fabricated by Torrey Pines Scientific. The controlled plate produces surface plate temperatures from −10 °C to 100 °C. These tests were performed at a surface plate temperature of 100 °C and a room temperature of 23 °C. The torch head of the pencil soldering iron used in this experiment is cylindrical with an internal and external radius of 2 mm and 8 mm, respectively. The pencil soldering is operated with butane gas, and the torch is equipped with the necessary elements for a good mixture of fuel and air.

## 5. Results and Discussion

Each ultrathin filter blocks 60% of light incident on it, and 40% of the light goes directly to its respective color channel. This percentage of incident light was selected to increase temperature resolution measurement. The Schlieren images are recorded in the digital camera’s red and blue color channel, and registered shadowgraph images are in the green color channel. Therefore, the optical system presented in this work record simultaneously Schlieren images sensitive to horizontal and vertical directions and shadowgraph images in one snapshot. Figure 2 shows that the crosstalk effect is presented mainly in the green color channel. Then, to improve shadowgraph image quality is needed to correct it against the crosstalk effect. The crosstalk coefficients can be determined in different ways; one way is directly from the overlapping between spectral intensity values of the color illumination source (*rgb* LED) and the spectral response on each digital camera pixel (see Figure 2). However, we opt for an experimental method to determine the crosstalk coefficients. The procedure consisted of directly illuminating the digital camera with each color of the LED. For each case, an image was recorded. Then, the contribution of each illumination color in each color channel of the camera was determined. The crosstalk coefficients correspond to the average intensity of each image color channel. Before calculating the crosstalk coefficients, the intensity of each LED color was equalized. The values obtained with this procedure were: *A_Rr_* = 6.55 × 10^4^, *A_Rg_* = 0, *A_Rb_* = 0, *A_Gr_* = 0.26 × 10^4^, *A_Gg_* = 5.78 × 10^4^, *A_Gb_* = 2.31 × 10^4^, *A_Br_* = 0, A*_Bg_* = 0, and *A_Bb_* = 6.55 × 10^4^. Once the crosstalk coefficients are known, the solution to Equation (8) is straightforward. These crosstalk coefficients are used to solve Equation (8) to obtain the values of *I_r_*, *I_g_*, and *I_b_*. *I_r_* and *I_b_* contain the Schlieren images, and the corrected shadowgraph image corresponds to *I_g_*.

### 5.1. Visualization Results

**Figure 3:**
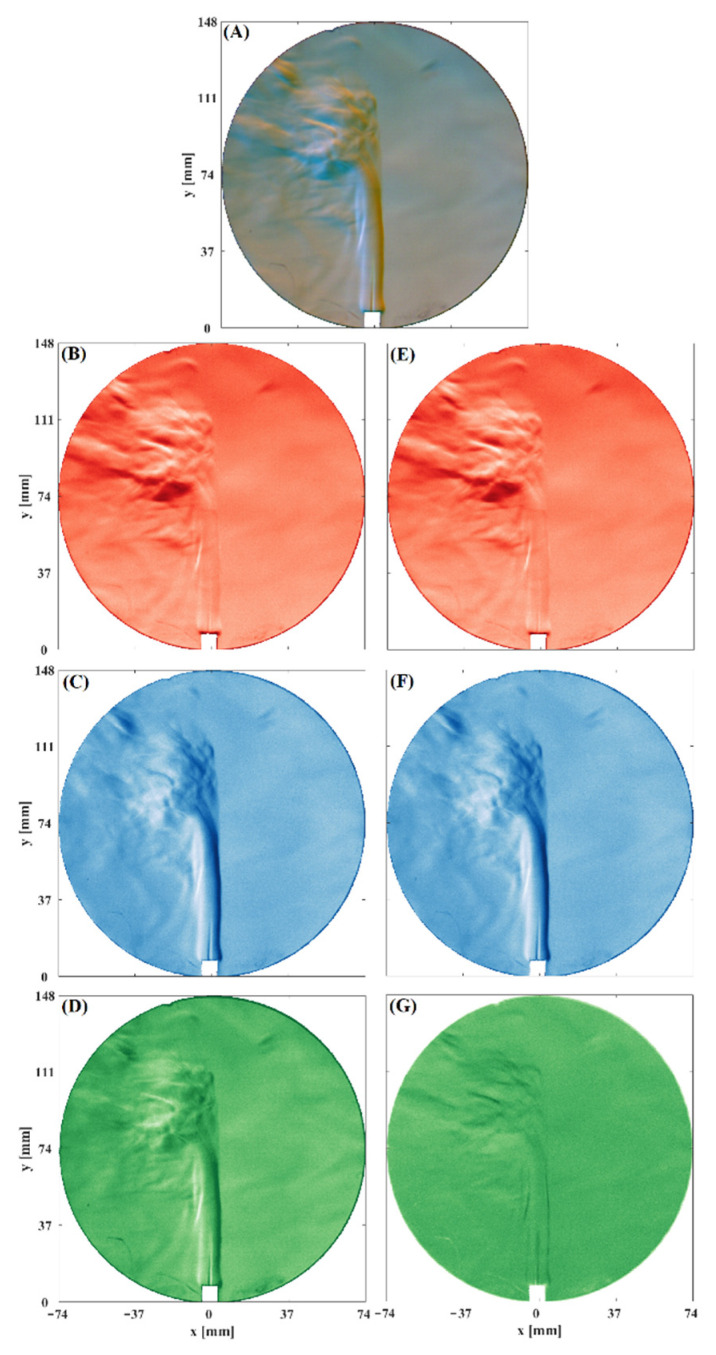
Flame and perturbed convective fluid flow of a pencil soldering iron. (**A**) *RGB* image. (**B**) Uncorrected horizontal sensitivity Schlieren image, (**C**) Uncorrected vertical sensitivity Schlieren image, (**D**) Uncorrected shadowgraph image, (**E**) Corrected horizontal sensitivity Schlieren image, (**F**) Corrected vertical sensitivity Schlieren image, (**G**) Corrected shadowgraph image.

This work tests the optical system by visualizing a pencil soldering iron flame. Figure 3 shows the image composed by each color channel of the digital camera of the fluid flow generated by the pencil soldering iron flame. Separating the images of each color channel, we can appreciate the contribution of both Schlieren images for this case. Moreover, components of the Schlieren effect can be observed in the shadowgraph image. Therefore, image correction is applied by solving Equation (8). The impact of this correction is notorious mainly in the shadowgraph image; it eliminates the Schlieren contribution leaving only the shades. On the other hand, the horizontal and vertical sensitive Schlieren images remain unchanged after applying the crosstalk correction. This last result is supported by what is shown in Figure 2, where the crosstalk occurs mainly in the green channel.

On the other hand, the images show the details of the fluid flow. Observe that each of the schlieren images complements the other. For example, in the regions where there are shadows, in its complementary image, it appears illuminated. In this way, with both Schlieren images, greater detail of the flows is achieved. In addition, the shades of the corrected shadowgraph images of the case under study represent the second derivative of the density flow. Indeed, these results show that the sensitivity of the optical system representing Schlieren’s results is higher than that of the shadowgraph optical system.

### 5.2. Temperature Fields Calculations

**Figure 4:**
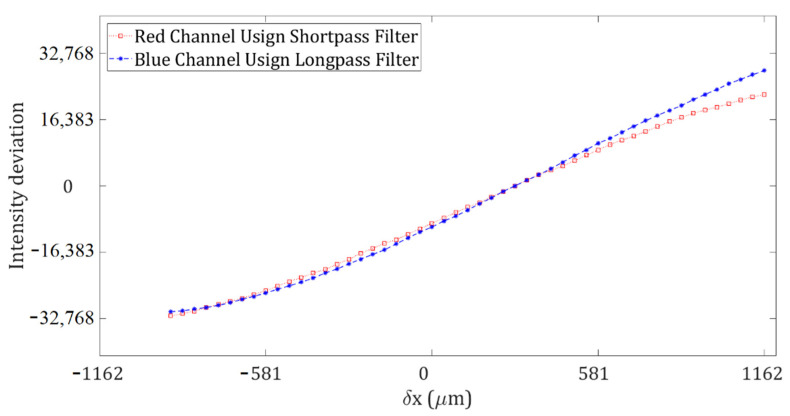
A calibration curve of the red and blue color channels for a particular pixel.

The temperature fields are measured using calibration curves. A calibration curve relates light intensity values of Schlieren images to ray angle deviation. Calibration curves are determined by moving the knife-edge (in our case, a filter) in fixed step sizes from the maximum and minimum light intensity. In each position, an image is registered. Calibration curves for each pixel were obtained by moving each filter laterally in its respective direction from −750 μm to +1162 μm with a step size of Δx = 37.5 μm. Figure 4 shows a calibration curve for a specific pixel of each color channel because of moving the filter. Note that each pixel of a color channel has its calibration curve. The light intensity deviation of each pixel of a Schlieren image is related to the closest value of the light deviation of its corresponding calibration curve.

The intensity deviation of a Schlieren image and a calibration curve are obtained by subtracting each one from the Schlieren image obtained at the reference position. The reference position represents the condition when the knife edge is at a value between the maximum and minimum light intensity, i.e., when the intensity value is about 40% of the light at the observation plane. Once the intensity deviations are converted to *δx* and *δy* values, a numerical integration algorithm is used to calculate *ρ* in Equation (3). Then, the value of *ρ* can be substituted into Equation (4) to obtain the corresponding temperature [27].

(9) $$T\;=\;\frac{\rho_{0}}{\rho }T_{0}\;=\;\frac{n_{0}-1}{n_{m}-1}T$$

In Equation (9) *n*_0_, and *ρ*_0_ are the refractive index and density at reference temperature *T*_0_, respectively, and *T* and *n_m_* are the temperature and refractive index of interest. A deep discussion about determining calibration curves to calculate temperature fields can be found in other works [8,9]. 

**Figure 5:**
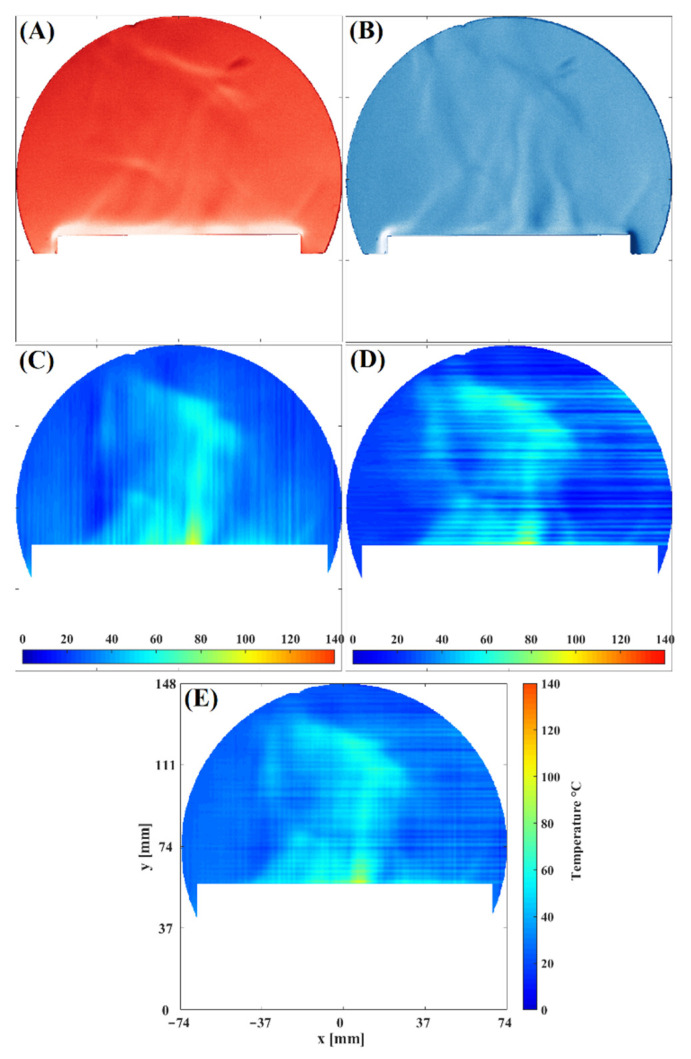
An instantaneous temperature field of the convective fluid flow generated by the plate. (**A**) vertical sensitivity Schlieren image, (**B**) horizontal sensitivity Schlieren image, (**C**) Temperature field of (**A**), (**D**) Temperature field of (**B**), (**E**) Combination of temperature field of (**C**,**D**).

The tests are achieved by measuring the temperature fields of the convective fluid flow generated by a heated Rectangular Metal Plate. The plate was placed in the work section with its width along the “z” axis in the experiments. For the analysis, we assume that close to the surface of the metal plate, where the fluid flow fluctuations are not so high, the behavior of the fluid can be considered nearly 2D [8,9]. Figure 5 shows Schlieren’s images that will be processed to determine temperature fields. Figure 5A,B show that the fluid flow pattern has refractive index variations in the horizontal and vertical directions. Therefore, these images provide a complete view of the fluid flow behavior. Moreover, this figure contains the temperature fields calculated after applying the procedure explained previously. Figure 5C,D correspond to temperature fields of Schlieren images sensitive to horizontal and vertical density variations directions, respectively. Notice that the fluid flow patterns in each image complement each other. Finally, to obtain the flow’s total temperature field, each result’s patterns were added to reconstruct the final temperature field (see Figure 5E). Observe that each temperature field contributes specific details to the final result. Consequently, the temperature value close to the surface of the plate is consistent with the temperature set at the programmable rectangular metal plate [8,9]. In addition, we can observe that the optical technique presented in this work enhances the detail of the convective fluid flow temperature fields

**Figure 6:**
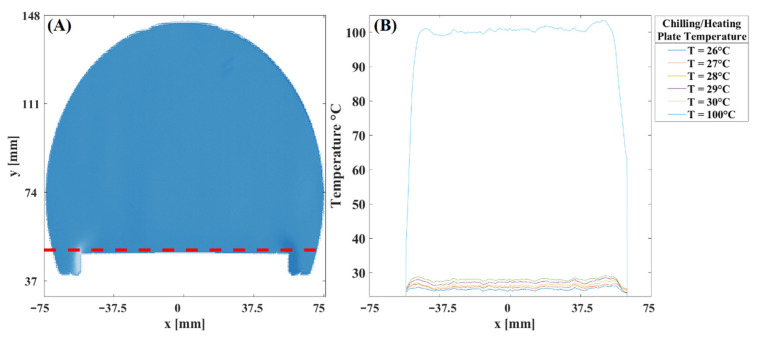
The temperature profile fields of the plate close to the surface. (**A**) Schlieren image at a temperature of 1.6 mm on the surface of the plate, (**B**) temperature profiles for the different temperatures of the rectangular plate.

*Temperature resolution of the optical system.* A controlled experiment to determine the sensitivity of the optical system was performed to determine the temperature resolution. Again, we used the rectangular plate for such a purpose. The metal plate was set to a temperature value of 26 °C up to 30 °C with a step size of 1 °C. Figure 6 shows a Schlieren image; on the Schlieren image, a dotted red line indicates the place used to calculate the temperature profiles for different temperature values. The intensity profile used for this calculation corresponds to a position of 1.6 mm from the surface of the metal plate. In this calculation, we used only Schlieren images with refractive index variations along the horizontal direction. Therefore, we can observe the symmetry in the shape of the profile characteristic of the Schlieren images. Note that the temperature profile of each Schlieren image is resolved correctly along the metal plate. The temperature profile in the figure was obtained by averaging the profiles of 25 Schlieren images. Finally, we overlapped the temperature profile of the case shown in Figure 5. Note that the results show a temperature profile almost constant along the rectangular plate. In addition, indeed, according to this figure, the system’s sensitivity is approximately one °C. The resolution of the optical system reported in this study was achieved because the knife edge was located in a reference position that allowed 40 percent of the incident light to pass through. This value was selected because it will enable us to increase the sensitivity without changing the camera parameters used in these experiments, i.e., exposure time, gain values, and light intensity levels of the camera.

## 6. Conclusions

We presented a method to visualize density variations in fluid flows. This optical system measured temperature fields using Schlieren images and a calibration procedure. A Schlieren setup was used for such a purpose. The optical system allows obtaining horizontal and vertical sensitivity Schlieren and shadowgraph images simultaneously. The results show that shadowgraph images have low sensitivity due to the inherent characteristics of the optical system. The advantage of the new approach lies in obtaining in a single image the refractive index variations in the horizontal and vertical directions and shadowgraph of the phenomenon studied. With this, it is possible to unite the characteristics not visualized with the standard Schlieren arrangement, resulting in improved visualization and measuring of temperature fields.

## Data Availability

Data underlying the results presented in this article are not publicly available but may be available from the corresponding authors upon reasonable request.
